# Teaching impact in pediatric minimal access surgery: Personal perspective from “Fellow”

**DOI:** 10.4103/0972-9941.28183

**Published:** 2006-12

**Authors:** Dragan Kravarusic

**Affiliations:** Alberta Children’s Hospital, Calgary, Alberta, Canada

**Keywords:** MAS, pediatric, teaching

## Abstract

The global objective of this paper is to review from the “Fellow” perspective, the current status of pediatric minimal access surgery (MAS) in terms of teaching feasibility, safety and impact on standard practice paradigms. In the pediatric general surgery field, surgeons are dealing with a wide range of pathology that includes thoracic, abdominal, urological and gynecological procedures. The learning curve is slow because of a relatively small volume of patients. However, gradually but steadily, a significant proportion of the procedures traditionally performed, with major open exposures at present, are preferentially performed by minimal access. Currently, minimal access surgery training is incorporated into adult general surgery residency/fellowship programs and teaching techniques of pediatric MAS are available only as seldom international workshops. Pediatric surgery fellowship programs with incorporated guidelines for MAS training are just recently feasible in select centers, mostly as “self” established programs. In many other pediatric surgery centers, teaching the “glamour” of MAS is quite dependent on a program director’s vision. Integration of MAS training into the secondary residency/fellowship curriculum of pediatric surgeons is the inevitable goal. MAS- minded education and research through adequate training will pay dividends and “manufacture” competent, contemporary trainees. National Pediatric Surgery Associations should be responsible for setting criteria that consider the MAS for accreditation with maintaining the international standards of these teaching programs.

## INTRODUCTION

Ever since its entry more than two decades ago, minimal access surgery (MAS) introduced a sweeping revolution in surgical practice. Worldwide, the volume of MAS procedures has rapidly increased in recent years and consequently, recent general surgery graduates are seeking MAS fellowships in record numbers. The field of pediatric surgery is no exception and there are numerous patients who can benefit from this approach.[1,2]

MAS is a natural extension of traditional surgical treatment, but the techniques and dexterity required to master these procedures are a separate set of skills. Currently, MAS training is incorporated into adult general surgery residency programs and MAS fellowship opportunities are feasible in well-established programs in many centers. In contrast, teaching techniques of pediatric MAS are rarely available as international course lectures (didactic or with animal models) or as broadcasting of specific case demonstrations used as teaching opportunities. Pediatric surgery fellowship programs with incorporated guidelines for MAS training are available only recently in select centers, mostly as “self” established programs. In many other pediatric surgery centers, teaching the “glamour” of MAS is quite dependent on a program director’s vision.

## MATERIALS AND METHODS

The global objective of this paper is to review from the “Fellow” perspective, the current status of pediatric minimal access surgery in terms of teaching feasibility, safety and impact on standard practice paradigms of a single institution. The Alberta Children’s Hospital is a pediatric tertiary care facility for patients from the neonatal period, to late adolescence. This report is a retrospective review of all MAS procedures performed in the pediatric general surgery department, from June 2004 to June 2005. The selection of patients and procedures in this series was highly individualized between staff surgeons with consideration regarding patient/parent preference, surgeon’s experience, complexity of the procedure and the patient’s medical condition. All surgeries were performed by the single pediatric surgery fellow, with close supervision by the attending staff surgeon. Absolute contraindications included hemodynamic instability, severe cardiac diseases, pulmonary insufficiency and malignancy.

## RESULTS

A total of 314 MAS procedures were performed in 311 patients, of which 56 were thoracoscopic and 258 laparoscopic [Table 1]. The range included 28 different procedures with an overall conversion rate of 3.5% (1.78% in thoracoscopic and 3.87% in laparoscopic procedures).

**Table 1:** The spectrum of MAS procedures with comparison to relevant conventional “open” procedures during the same period of time.

		MAS	OPEN

		Thoracoscopic	
Type of procedure	**No. Conversions**		
Lung biopsy/wedge	18	0	4
Mediastinal biopsy	4	1	0
Drainage and debridement for empyema	11	0	0
Excisions of benign tumor/cyst	2	0	0
Aortopexy	1	0	2
Blebectomy for recurrent pneumothorax	6	0	0
Nuss operation	14	0	0
	**Laparoscopic**		
Appendectomy	56	4	242
Cholecystectomy	24	0	0
Splenectomy	4	1	1
Fundoplication	42	0	7
Heller myotomy	4	0	0
Pyloromyotomy	3	0	12
Adhesiolysis	8	1	14
Gastrostomy	31	0	15
Cecostomy	6	1	1
Excision of mesenteric cystic mass	1	0	1
Liver biopsy	2	0	2
Excision of urachal cyst	1	0	3
Retroperitoneal biopsy/lymph node			
sampling	2	0	6
Bowel resection’s for Crohn’s/FAP	6	2	5
Pull through for Imperforated anus	2	1	4
High ligation for varicocele	12	0	0
Fowler-stephens operation 1^st^ stage	18	0	0
Exploration for contralateral hernia	22	0	0
Ovarian cyst drainage/resection	9	0	0
Ovarian detorsion	1	0	0
Nephrectomy	4	0	2
Total	314	11	321

MAS: Minimal access surgery

The great majority (90.32%) of relevant thoracic procedures were performed thoracoscopically. Lung biopsy was the most common performed procedure (18 cases) with excellent results and a zero conversion rate. Minimal access repair of pectus excavatum (nuss operation) was performed in 14 children with good cosmetic results, without complications and conversions. Drainage of empyema with debridement was done in 11 cases and this modality is especially suitable for children where general anesthesia is unavoidable. Thoracoscopic aortopexy was very challenging, but the low volume of these procedures prevents us from reading valid conclusions of this approach. For solitary, simple, cystic lung lesions, the MAS approach seems to be a very comfortable and safe option.

From selected routine abdominal surgery, laparoscopic procedures replaced open surgery in variable percentage (45%). (In total, 258 from 573 procedures.) All 24 cholecystectomies were performed as laparoscopic (100%) and of the 49 fundoplications, 42 were performed laparoscopically (85.71%). These two procedures have already become the “gold standard” in pediatric surgery practice. The insertion of gastrostomy tube only or as part of anti-reflux procedures for patients with feeding difficulties, was done in 31 cases. This technique provided very good visualization and was especially important for choosing “the right place” for a G-tube. Of the 5 splenectomy cases, 4 were done by laparoscopy (80.0%), with 1 open evacuation of the large spleen. During this period of time, 298 appendectomies were performed, of which 56 were done by laparoscopy (18.79%), with 4 conversions (7.14%) for perforated appendicitis with diffuse purulent peritonitis. Selection for laparoscopic appendectomy was focused to a certain group of patients (obesity, female adolescents and patients with uncertain diagnosis) who were especially suitable for such a modality. Laparoscopic cecostomy for bowel management of incontinent or severely constipated patients (mostly spina bifida group) were performed in 6 cases. This approach is very well-established at this hospital, with long term follow-up and documentation of improved quality of life. From six laparoscopic bowel resections (4 for Crohn’s disease and 2 total colectomies for familial adenomatous polyposis syndrome) with extracorporal anastomosis, we had conversion in 2 cases (33.3%) due to insufficient visualization and probably lack of experience. However, the general departmental approach is to continue with performing these procedures in selected, suitable patients, to overcome pitfalls and maintain progress in MAS. Laparoscopic pull-through for imperforate anus was done in two cases, after diverting colostomy was performed in the postnatal period. One case was converted after a time- consuming attempt to release severe adhesions, in a child who previously had a V-P shunt for hydrocephalus. The other child had an uneventful surgery and this result encouraged us to continue with this modality in suitable cases of high imperforate anus with fistula. Laparoscopic high ligation for varicocele (12 cases) and Fowler-Stephens operation (18 cases) for undescended, intra-abdominal testicle, were routine MAS procedures that replaced almost all “open” approaches for such pathology. From the so-called “gynecological procedures”, all cases were done as “urgent” cases of acute abdominal pain with peritoneal signs and uncertain diagnosis. Nine cases of ovarian cysts (ruptured or hemorrhagic) and one case of ovarian torsion were successfully treated in a timely manner. Laparoscopic hand-assisted bilateral nephrectomy performed for persistent post transplant polyuria in 2 patients with juvenile nephronophthisis was safe, was tolerated very well and allowed out-patient follow-up of these patients who were otherwise dependent on intravenous infusion.

## DISCUSSION

Various workshops have been developed in adult MAS training programs. Computer-generated virtual reality systems allow sensory interaction and provide “hand-eye coordination” models which are especially useful for self-assessment in simulation-based surgical skills training.[3,4] The reliability and validity of this modality in teaching has been confirmed in numerous studies.[5–8] MAS in animal laboratories has been recognized as well as a method of teaching, developing and refining surgical techniques which contribute to a beneficial outcome in patients.[9] However, pediatric MAS workshops are quite rare and attendance at a 1 or 2 day workshop does not translate into expert practice and is not sufficient to be considered as credentialing activity. So the only way to reach competency in pediatric MAS, is the acquisition and safe performance of sufficient volume and a range of relevant procedures. An operation can be reduced to its component steps, which can be learned and mastered over a number of separate operations on different patients.[10] Supervised operating with structured objective assessment and feedback remains an essential part of surgical training, because it includes all of the variables encountered in surgery.[11,12]

The intent of this discussion is to acquaint how important it is to teach trainees, what can and should be done via MAS in the pediatric population. Today’s parents are bringing their children in for surgical consultation after profound “search” on the internet for a specific problem. They are well informed about therapeutic options and require the best possible medical care. An important issue that must be kept in mind, is that just because a procedure can be done technically, does not mean that it is better for the patient. The MAS technique must be at the very least and as safe and effective as the procedure it purports to replace.

The surgeon must be fluent with conventional surgical methods in situations when the MAS approach is not feasible or results in problems that require rapid conversion to “open” operation. Nevertheless, there is a learning curve and the potential for a higher complication rate is one of the most uncomfortable facts that teaching staff have to face, especially when this is judged against the excellent results of many pediatric procedures achieved by open surgery. Some senior pediatric surgeons are reluctant to promote MAS skills when they are already highly proficient in open surgery.

Without open-minded and supporting staff/consultants as leaders, surgical residents/fellows are unable to gain skills and make any progress, unless every potential MAS procedure is evaluated. MAS should be appraised not merely on its feasibility or by the enthusiasm or euphoria of personal ego or achievement, but rather as a pragmatic clinical teaching process, as it applies to the pediatric population.

The scene is fast moving and only encouraging programs with established training of MAS techniques will “manufacture” competent, contemporary trainees who are under close supervision of their teachers, through education and research, constantly challenging the order of criterions for MAS procedures in modern surgery. This is especially important in the pediatric general surgery field where surgeons are dealing with a wide range of pathology that include thoracic, abdominal, urological and gynecological procedures, where the learning curve is slower because of the relatively small volume of patients. Adequate training will pay dividends and national pediatric surgery associations should be responsible for setting criteria that consider MAS for accreditation with maintaining international standards of teaching programs and courses.

Integration of MAS training into the secondary residency/fellowship curriculum of pediatric surgeons is the inevitable goal. Interaction through International Pediatric MAS groups is very welcome among trainees and hopefully multi-institutional prospective studies will allow determination of standard guidelines for MAS teaching in the pediatric population.
